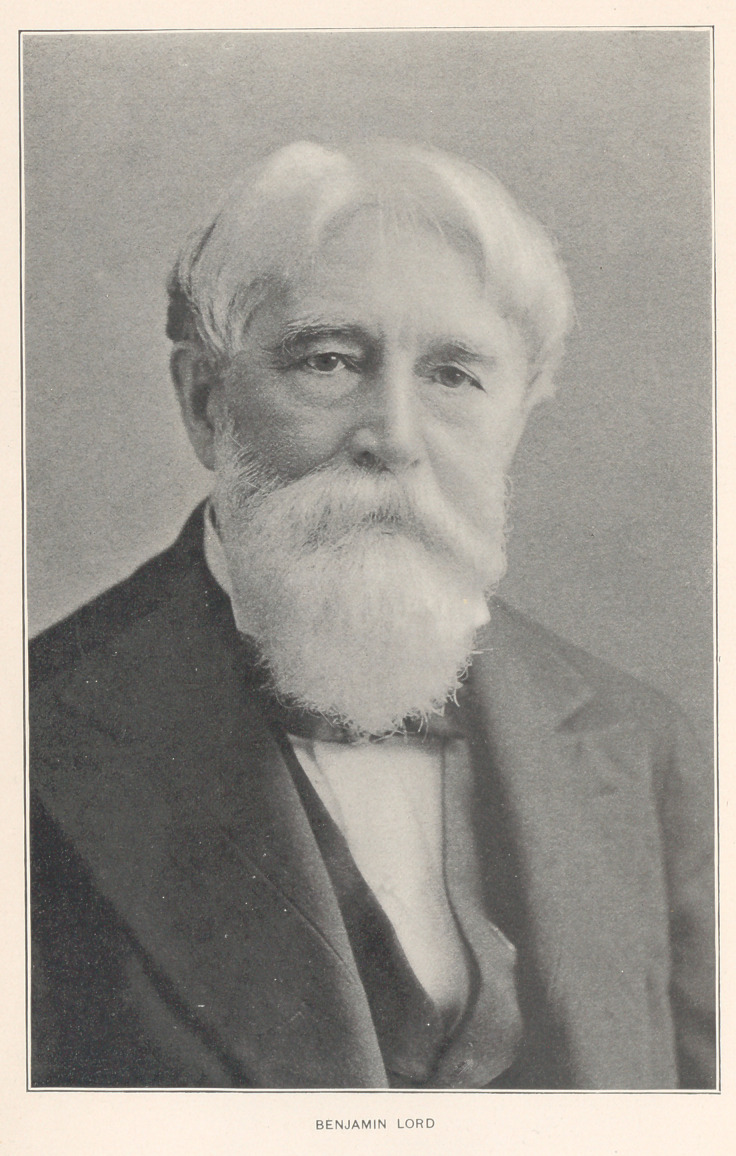# Dr. Benjamin Lord

**Published:** 1902-07

**Authors:** 


### Biographical Sketch.

### DR. BENJAMIN LORD.

THE recent death of Dr. Benjamin Lord, May 3, 1902, leaves another vacant place in the ranks of those who made dentistry what it was in the nineteenth century, and who, by their work, laid the foundation for a future superstructure worthy the great advance that should be made in the next one hundred years.

Dr. Lord, although a quiet, unassuming man, possessed a power that probably only his most intimate friends suspected. He was one who truly hid his light under a bushel, but it was always burning and ready for those emergencies that came to him oftener than to most men.

Dr. Lord was born on a farm near Trenton, N. J., August 25, 1819. The portion of his life from then to maturity was probably spent, as most farmer's sons spend theirs, in hard work and little relaxation, but not much is known by the writer of this period in his life. It is very evident that the trying and monotonous labor of the farm failed to meet his taste or his ambitions, for we find him prior to 1840 in Newark, N. J., in the office of his brother, William G. Lord, studying dentistry. He began practice for himself in 1840, in Monticello, Sullivan County, N. Y., but soon abandoned this and moved to New York City.

He rented an office here in a boarding-house on Broadway below Tenth Street. The prejudice then existing in the average medical mind against any association with dentistry is well illustrated by an incident occurring here, which forced Dr. Lord to give up this office. A physician boarding in the family objected to a dentist's presence in the house, and for the sake of peace he removed to the New York University Building, on Washington Square. This has within a few years been replaced by a large business building.

Dr. Lord's studious character led him to the study of medicine, which doubtless would have ended in his receiving the degree, but after taking one year at the Medical College of the New York University, he was obliged to abandon the idea on account of increasing practice, which necessarily absorbed all his time.

The writer's intimacy with Dr. Lord fills nearly a third of a century. He then lived in a home well fitting the man and his very large practice, and in this he remained to the day of his death. Notwithstanding the great change from the simple farm life of his youth, he ever remained the quiet, humble worker, active always in good works.

His methods of practice continued as he was taught, and he belonged to that fast-disappearing race of dentists who practised during the first decade of the second half of the nineteenth century. To say that he made no changes in his practice would be doing Dr. Lord great injustice. While he never adapted himself to modern appliances, he was constantly devising instruments to facilitate his methods of work, and was exceedingly generous in distributing these among his professional friends. Some of these are of very great value in the manipulation of non-cohesive gold and in the finishing of fillings on proximate surfaces.

He possessed remarkable tactical sense, and was in the habit, even in recent years, of filling and refilling cavities in extracted teeth to test different kinds of gold- and tin-foil and also various shapes of instrument points.

It was in his interest in local society work and in dental literature that Dr. Lord found his chief interest outside of philanthropic labors and his daily practice. He was untiring in his efforts in maintaining an advanced standard for his profession. He regarded with disfavor the constant attempts of the commercial interests to dominate dentistry. To him all connection with trade was unprofessional. In furtherance of this view he became an active supporter of the INTERNATIONAL DENTAL JOURNAL, contributed freely of his time and means to advance its circulation, and retained his active interest in it up to the day of his death.

In the local dental organizations with which he was at various times connected he was always active, and contributed papers from time to time of great practical value. He was not, however, an active disputant, and seemed to the writer to have a preference to allow others to do this work, all the more, perhaps, as the trend of modern dental thought was outside of the channels he was accustomed to travel. Notwithstanding this conservative tendency of his mind, he possessed a liberal spirit, always willing to allow to others the freedom of opinion he demanded for himself.

He was deeply interested in benevolent and church work. His nature was essentially religious, and this, coupled with a thorough sympathy with the sufferings of the poor and those in need of help for other reasons, led him into charitable labor in the various institutions of New York. For most of his life he was a member and active worker in the Methodist Episcopal Church, but in recent years he became identified with the Reformed Episcopal Church.

A long life of active professional labor, combined with an excellent business capacity, enabled him to accumulate a considerable property. The exact disposition of this is unknown to the writer, but it is understood he left a large proportion of his means to church and charitable institutions.

The dental profession has lost in Dr. Lord one of its most valued members and the general public one of its truest men, one always earnest for the amelioration of the ills of humanity and ever ready to blaze a path in the tangled mazes of a world's life for those who had failed to reap the rewards that had come to him. Reverently we lay open this noble life of our friend and colleague to the consideration of the younger generation of dentists that they may walk in his path and reap the reward that belongs always to the earnest and true-hearted in all the vocations of life.

## Figures and Tables

**Figure f1:**